# Association of Sjögrens Syndrome in Patients with Chronic Hepatitis Virus Infection: A Population-Based Analysis

**DOI:** 10.1371/journal.pone.0161958

**Published:** 2016-08-25

**Authors:** Chih-Ching Yeh, Wen-Chang Wang, Chien-Sheng Wu, Fung-Chang Sung, Chien-Tien Su, Ying-Hua Shieh, Shih-Ni Chang, Fu-Hsiung Su

**Affiliations:** 1 School of Public Health, College of Public Health and Nutrition, Taipei Medical University, Taipei, Taiwan; 2 Department of Public Health, China Medical University, Taichung, Taiwan; 3 The Ph.D. Program for Translational Medicine, College of Medical Science and Technology, Taipei Medical University, Taipei, Taiwan; 4 Division of Allergy, Immunology, and Rheumatology, Far Eastern Memorial Hospital, Taipei, Taiwan; 5 Department of Health Services Administration, College of Public Health, China Medical University, Taichung, Taiwan; 6 Faculty of Public Health, Mahidol University, Bangkok, Thailand; 7 Department of Family Medicine, Taipei Medical University Hospital, Taipei, Taiwan; 8 Department of Family Medicine, School of Medicine, Taipei Medical University, Taipei, Taiwan; 9 Department of Family Medicine, Taipei Medical University, Wan Fang Hospital, Taipei, Taiwan; 10 Management Office for Health Data, China Medical University Hospital, Taichung, Taiwan; 11 Master Program in Long-Term Care, College of Nursing, Taipei Medical University, Taipei, Taiwan; 12 School of Medicine, Flinders University, Bedford Park, Australia; Centre de Recherche en Cancerologie de Lyon, FRANCE

## Abstract

**Objective:**

The association between Sjögren’s syndrome (SS) and chronic hepatitis virus infection is inconclusive. Hepatitis B (HBV) and hepatitis C virus (HCV) infections are highly prevalent in Taiwan. We used a population-based case-control study to evaluate the associations between SS and HBV and HCV infections.

**Materials and Methods:**

We identified 9,629 SS patients without other concomitant autoimmune diseases and 38,516 sex- and age-matched controls without SS from the Taiwan National Health Insurance claims data between 2000 and 2011. We utilized multivariate logistic regression to estimate the odds ratios (ORs) and 95% confidence intervals (CIs) of the associations between SS and HBV and HCV infections. Sex- and age-specific (<55 and ≥55 years) risks of SS were evaluated.

**Results:**

The risk of SS was higher in patients with HCV than in those without chronic viral hepatitis (OR = 2.49, 95% CI = 2.16–2.86). Conversely, HBV infection was not associated with SS (OR = 1.10, 95% CI = 0.98–1.24). Younger HCV patients were at a higher risk for SS (<55 years: OR = 3.37, 95% CI = 2.62–4.35; ≥55 years: OR = 2.20, 95% CI = 1.84–2.62). Men with HCV were at a greater risk for SS (women: OR = 2.26, 95% CI = 1.94–2.63; men: OR = 4.22, 95% CI = 2.90–6.16). Only men with chronic HBV exhibited a higher risk of SS (OR = 1.61, 95% CI = 1.21–2.14).

**Conclusion:**

HCV infection was associated with SS; however, HBV only associated with SS in men.

## Introduction

Sjögren’s syndrome (SS) is a chronic autoimmune disorder which results in the lymphocytic infiltration of the exocrine glands and polyclonal B-cell activation [1]. Dryness of the mouth and eyes are common clinical manifestations. The female:male ratio in the prevalence of SS reported in literature varies geographically from 8:1 to 14:1 [2–5]. The average age of SS patients is between 53 and 56 [2,6].

The etiology of SS is thought to arise from a specific combination of individual environmental and genetically predisposed factors. Viruses such as hepatitis C virus (HCV) are considered to be exogenous risk factors for developing the disease [7]. Since the earliest report in 1992 by Haddad et al., numerous authors have reported a relationship between SS and HCV in the past two decades [6,8–13]. However, the pathogenesis of HCV-associated SS is not well established.

While there is evidence demonstrating the role of HCV infection in the etiology of SS-like conditions, there are conflicting reports regarding the nature of the association between SS and chronic hepatitis B virus (HBV) [8,14–17]. On account of the lower prevalence of the hepatitis B core antibody in patients with autoimmune diseases, such as SS, one study proposed that HBV infection may confer some degree of protection against autoimmune disorders [14]. Patients with SS have been reported to have a prevalence of chronic HBV infection of 0.83%, which is similar to that of the general population in Spain (0.7%). This suggests that chronic HBV infection may not be associated with SS in that region, with an SS–HBV/SS–HCV ratio of 1:10 [15].

Taiwan is an endemic area for HBV and HCV infection [18]. While this may suggest that virus-related autoimmune diseases pose a threat to public health in Taiwan, no study to date has investigated the prevalence of SS among chronic HBV carriers. Therefore, this study set out to estimate that prevalence using a case-control study and population-based dataset.

## Material and Methods

### Data sources

We designed a population-based case-control study using the National Health Insurance Research Database (NHIRD). This database consists of data sourced from the National Health Insurance (NHI) program. Taiwan instituted this program in 1995 to meet the needs of providing affordable health care for the entire population [19]. By the end of 2004, this compulsory single-payer insurance program covered approximately 99% of the Taiwan’s total 23 million person population [20].

We selected our control subjects from the Longitudinal Health Insurance Database 2000 (LHID2000) which contains all the original claims data for one million participants who were randomly extracted from the Taiwan NHI program in the year 2000. There are no statistically significant differences in age, sex, or health care costs between the LHID and NHI enrollees [21].

We selected our cases from the Registry for Catastrophic Illness Patient (RCIP) database. Taiwanese patients who have contracted a life-altering and debilitating disease are eligible to apply for inclusion in the RCIP database to obtain significant discounts on treatment. SS (ICD-9-CM 710.2) is an NHI-defined catastrophic illnesses [22]. Consideration for a Catastrophic Illness Certificate (CIC) for SS is based on the 2002 European classification criteria for SS [23] and requires the submission and review of relevant clinical and laboratory information as well as a review by a panel of rheumatologists commissioned by the Bureau of the NHI system. Therefore, the RCIP data are highly accurate and reliable and have been used for several prior studies on SS [3,24–26]. The details of the program have been well documented in previous papers [27]. This study was deemed exempt from full review by the International Review Board of China Medical University and Hospital Research Ethics Committee (IRB permit number: CMU-REC-101-012). Patient informed consent was not required for this study as the NHI anonymizes the patient identification data prior to its release to the public.

### Study sample

We first identified patients from the RCIP database with newly diagnosed SS (International Classification of Diseases, Ninth Revision, Clinical Modification [ICD-9-CM] 710.2) between 2000 and 2011. Patients with secondary SS, which is defined by the presence of diffuse diseases of connective tissue (ICD-9-CM 710.X except 710.2) and rheumatoid arthritis (ICD-9-CM 714.0), were excluded from this study.

Our purpose in this investigation was to estimate how chronic hepatitis B (ICD-9-CM 070.2, 070.3, and V02.61) and hepatitis C (ICD-9-CM 070.41, 070.44, 070.51, 070.54, and V02.62) infections associate with SS. For HBV infection, HBsAg was the primary serum marker. We also excluded patients with a history of HIV (ICD-9-CM 042, 043, 044, and V08) to avoid including patients with an occult HBV infection (the persistence of HBV in HBsAg-negative carriers) among co-infected patients [28]. After excluding 4,448 patients with secondary SS, 5 patients with HIV, and 11 patients with missing age- and sex-related information, we enrolled 9,629 patients with SS in this study.

**Fig 1:**
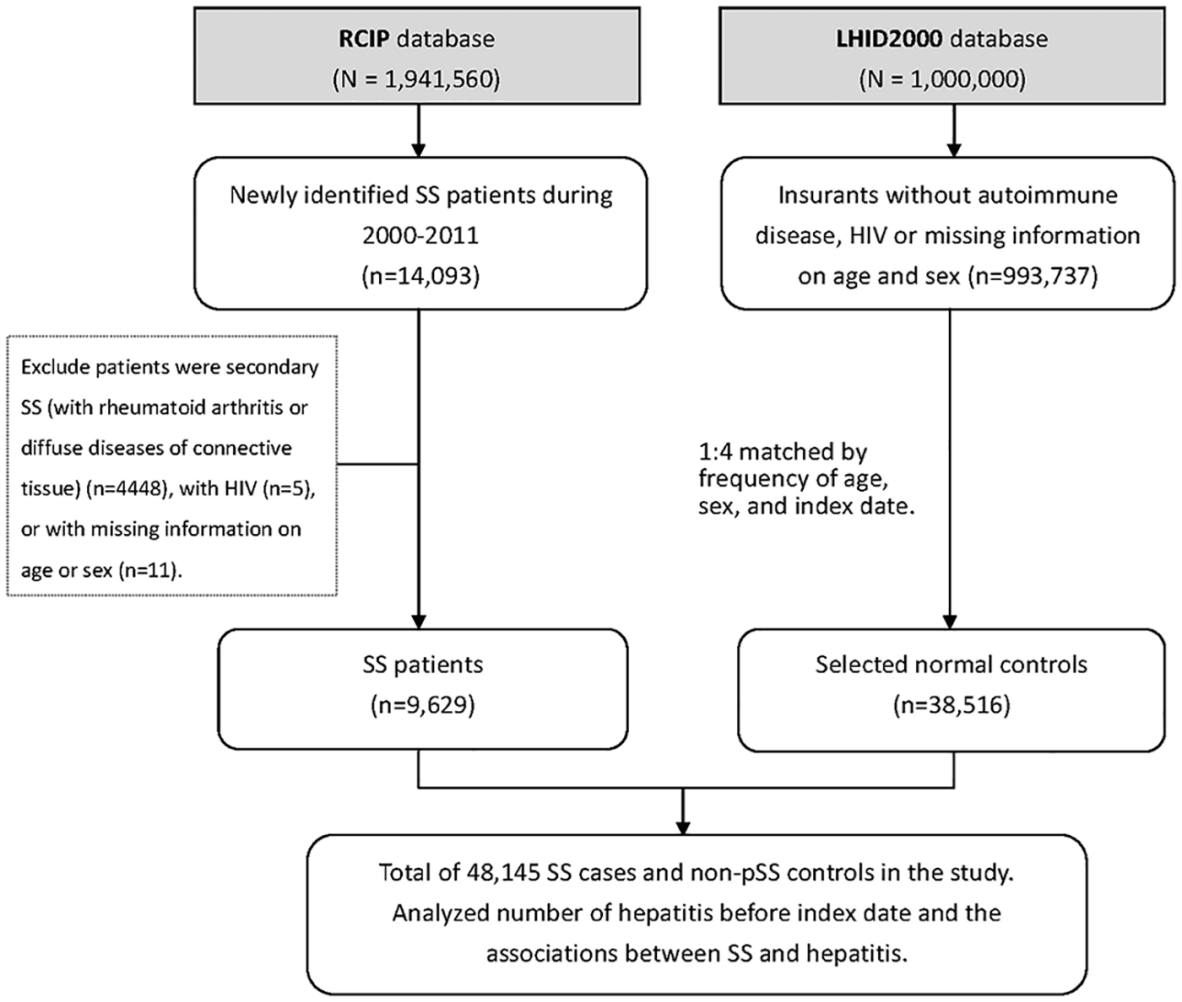
Study subject selection. The Sjögren’s syndrome (SS) patients were selected from the Registry for Catastrophic Illness Patient (RCIP) database and controls were selected from the Longitudinal Health Insurance Database 2000 (LHID2000) database in Taiwan.

The control group was composed of randomly selected age- and sex-matched people without a history of SS. We used SAS code PROC SURVEYSELECT to perform the random extraction of controls. The control-to-case ratio was 4:1. We calculated the age of each study by subtracting the date of birth from the index date. After the exclusion of 4,874 subjects with SS from secondary origins or other autoimmune diseases, 1,342 subjects with HIV, and 47 subjects with missing age- and sex-related information, 993,737 subjects were eligible control subjects. Finally, 38,516 controls were enrolled in this study. Fig 1 presents the subject recruitment for SS patients from the RCIP database and controls from the LHID2000 database in Taiwan.

### Statistical analysis

We used chi-square tests to compare the distributions of demographic characteristics, such as sex, age, occupation, monthly income, geographic location and urbanization level of the subject’s area of residence, and comorbidities between patients with SS and control subjects. We selected New Taiwan dollar (NT$) amounts of NT$15,840 and NT $25,000 as monthly income cutoff points. In addition, baseline comorbidities, such as diabetes mellitus, hyperlipidemia, hypertension, cancer, liver cirrhosis, renal disease, coronary artery disease (CAD), and chronic obstructive pulmonary disease (COPD), developed since 2000 were ascertained in subjects with and without SS by using diagnostic codes. This study only considered subjects to have HBV and HCV if they received at least three out-patient diagnoses or at least one in-patient diagnosis. We applied the same criteria to the detection of comorbidities. In our multivariate logistic regression analysis we adjusted for those variables that were found by the chi-squared test to differ significantly between SS patients and controls and provided odds ratios (ORs) and 95% confidence intervals (CIs) as measures of association for viral hepatitis and SS. In addition, sex- and age-specific ORs of SS associated with viral hepatitis were examined. We used SAS statistical software (Version 9.4 for Windows; SAS Institute, Inc., Cary, NC, USA) to perform all the statistical analyses conducted in this study. The significance level was set at 0.05.

## Results

### Characteristics of the study subjects

Table 1 lists the demographic characteristics of the SS (N = 9,629) and control groups (N = 38,536) in 2000–2011. Most subjects were women (87.0% in both groups). The female:male ratio in prevalence was 87:13 (6.7:1) and median age of the patients was 55 years. Patients with SS were more likely to live in Central Taiwan (27.3% vs. 19.8%, p < 0.0001), have blue-collar occupations (45.3% vs. 38.9%, p < 0.0001), and have lower incomes (43.8% vs. 30.5%, p < 0.0001) compared with the controls. In addition, patients with SS were more likely to have hyperlipidemia, cancer, liver cirrhosis, renal disease, CAD, and COPD.

**Table 1:** Demographic characteristics of Sjögren’s syndrome patients and control subjects for 2000–2011.

	Sjögren’s syndrome				
	No (N = 38,516)		Yes (N = 9,629)		
Variables	N	(%)	N	(%)	p value*
Sex					1.000
Women	33,516	(87.0)	8,379	(87.0)	
Men	5,000	(13.0)	1,250	(13.0)	
Age, years					0.026
<55	19,348	(50.2)	4,715	(49.0)	
≥55	19,168	(49.8)	4,914	(51.0)	
Geographic region					<0.0001
North	17,428	(45.2)	3,664	(38.1)	
Central	7,642	(19.8)	2,632	(27.3)	
South	10,233	(26.6)	2,584	(26.8)	
East and Islands	3,212	(8.3)	749	(7.8)	
Occupation					<0.0001
White collar	18,741	(48.7)	2,413	(35.1)	
Blue collar	15,000	(38.9)	3,117	(45.3)	
Retired and others	4,775	(12.4)	1,352	(19.6)	
Urbanization level					0.161
Urban	11,877	(30.8)	3,017	(31.3)	
Suburban	17,922	(46.5)	4,414	(45.8)	
Rural	8,717	(22.6)	2,198	(22.8)	
Monthly income, NT$					<0.0001
<15,840	11,737	(30.5)	4,218	(43.8)	
15,841–25,000	19,977	(51.9)	2,970	(30.8)	
≥25,001	6,802	(17.7)	2,441	(25.4)	
Comorbidities					
Diabetes	6,287	(16.3)	1,585	(16.5)	0.744
Hyperlipidemia	9,541	(24.8)	2,859	(29.7)	<0.001
Hypertension	13,525	(35.1)	3,410	(35.4)	0.583
Cancer	1,249	(3.2)	394	(4.1)	<0.001
Liver cirrhosis	409	(1.1)	263	(2.7)	<0.001
Renal disease	2,556	(6.6)	1,108	(11.5)	<0.001
Coronary artery disease	6,792	(17.6)	2,309	(24.0)	<0.001
Chronic obstructive pulmonary disease	10,870	(28.2)	3,976	(41.3)	<0.001

*Chi-square test

### Overall risk of SS with chronic viral hepatitis

Logistic regression analysis revealed that unlike chronic HBV, chronic HCV was significantly associated with the risk of SS (OR = 2.49, 95% CI = 2.16–2.86) after adjusting for the significant variables in Table 1 (Table 2). Patients with hyperlipidemia, liver cirrhosis, renal disease, CAD, and COPD were significantly associated with an increased risk of SS (OR = 1.14, 95% CI = 1.08–1.21;OR = 1.90, 95% CI = 1.60–2.26; OR = 1.55, 95% CI = 1.43–1.68; OR = 1.24, 95% CI = 1.17–1.33; OR = 1.68, 95% CI = 1.60–1.77 respectively).

**Table 2:** Comparison of hepatitis prevalence between Sjögren’s syndrome patients and control subjects for 2000–2011.

	Sjögren’s syndrome					
	No (N = 38,516)		Yes (N = 9,629)			
	N	(%)	N	(%)	Crude OR (95% CI)	OR (95% CI)^a^
Viral hepatitis B & C						
No	36,342	(94.4)	8,743	(90.8)	1.00 (reference)	1.00 (reference)
HBV	1,378	(3.6)	415	(4.3)	1.25 (1.12–1.40)^‡^	1.10 (0.98–1.24)
HCV	547	(1.4)	397	(4.1)	3.01 (2.65–3.44)^‡^	2.49 (2.16–2.86)^‡^
HBV+HCV	249	(0.7)	74	(0.8)	1.24 (0.95–1.60)^‡^	0.97 (0.74–1.28)
Comorbidities						
Diabetes	6,287	(16.3)	1,585	(16.5)	1.01 (0.95–1.07)	—
Hyperlipidemia	9,541	(24.8)	2,859	(29.7)	1.28 (1.22–1.35)^‡^	1.14 (1.08–1.21)^‡^
Hypertension	13,525	(35.1)	3,410	(35.4)	1.01 (0.97–1.06)	—
Cancer	1,249	(3.2)	394	(4.1)	1.27 (1.13–1.43)^‡^	1.13 (1.00–1.27)
Liver cirrhosis	409	(1.1)	263	(2.7)	2.62 (2.24–3.06)^‡^	1.90 (1.60–2.26)^‡^
Renal disease	2,556	(6.6)	1,108	(11.5)	1.83 (1.70–1.97)^‡^	1.55 (1.43–1.68)^‡^
Coronary artery disease	6,792	(17.6)	2,309	(24.0)	1.47 (1.40–1.56)^‡^	1.24 (1.17–1.33)^‡^
Chronic obstructive pulmonary disease	10,870	(28.2)	3,976	(41.3)	1.79 (1.71–1.87)^‡^	1.68 (1.60–1.77)^‡^

^a^Adjusted for age, sex, geographic region, occupation, monthly income, hyperlipidemia, cancer, renal disease, coronary artery disease, chronic obstructive pulmonary disease, and liver cirrhosis

* p<0.05

^†^ p<0.01

^‡^ p<0.0001

### Age-specific risk of SS with chronic viral hepatitis

Age stratification is based on the median age of the SS patients (<55 and ≥55 years). In Table 3, our model revealed that HCV patients aged <55 years had a 3.37-fold higher risk of SS (OR = 3.37, 95% CI = 2.62–4.35) than control patients while HCV patients aged ≥ 55 years had a 2.20-fold higher risk of SS (OR = 2.20, 95% CI = 1.84–2.62) than controls. In contrast, age did not play a role in the risk of SS among patients with HBV infections. The interaction between HCV status and the age (<55 and ≥55 years) for developing SS was statistically significant (p < 0.001).

**Table 3:** Odds ratios and 95% confidence intervals of Sjögren’s syndrome associated with chronic viral hepatitis according to age.

	Age < 55 years			Age ≥ 55 years		
Viral hepatitis B & C	Cases	Controls	OR (95% CI)^a^	Cases	Controls	OR (95% CI)^a^
No	4,332	18,368	1.00 (reference)	4,411	17,974	1.00 (reference)
HBV	220	761	1.05 (0.89–1.23)	195	617	1.12 (0.94–1.34)
HCV	140	133	3.37 (2.62–4.35)^‡^	257	414	2.20 (1.84–2.62)^‡^
HBV+HCV	23	86	0.77 (0.47–1.27)	51	163	1.08 (0.76–1.52)

^a^Adjusted for sex, geographic region, occupation, monthly income, hyperlipidemia, cancer, renal disease, coronary artery disease, chronic obstructive pulmonary disease, and liver cirrhosis

* p<0.05

^†^ p<0.01

^‡^ p<0.0001

### Sex-specific association between SS and chronic hepatitis

Table 4 lists the sex-specific ORs of SS for the chronic HBV and HCV groups compared with the non-hepatitis group. The sex-specific ORs of SS risk for HCV were 2.26 (95% CI = 1.94–2.63) for women and 4.22 (95% CI = 2.90–6.16) for men. The effect of the interaction between HCV status and sex on the risk of SS was statistically significant (p < 0.001). By contrast, the sex-specific ORs of SS were higher in men with a sole HBV infection (OR = 1.61, 95% CI = 1.21–2.14).

**Table 4:** Odds ratios and 95% confidence intervals of Sjögren’s syndrome associated with chronic viral hepatitis according to sex.

	Women			Men		
Viral hepatitis B & C	Cases	Controls	OR (95% CI)^a^	Cases	Controls	OR (95% CI)^a^
No	7,652	31,652	1.00 (reference)	1,091	4,690	1.00 (reference)
HBV	337	1,164	1.06 (0.93–1.20)	78	214	1.61 (1.21–2.14)^†^
HCV	330	486	2.26 (1.94–2.63)^‡^	67	61	4.22 (2.90–6.16)^‡^
HBV+HCV	60	214	0.90 (0.66–1.22)	14	35	1.42 (0.74–2.73)

^a^Adjusted for age, geographic region, occupation, monthly income, hyperlipidemia, cancer, renal disease, coronary artery disease, chronic obstructive pulmonary disease, and liver cirrhosis

* p<0.05

^†^ p<0.01

^‡^ p<0.0001

### Interaction between sole chronic HCV infection and comorbidities (DM, CAD and liver cirrhosis) on risks of SS

Since some comorbidities have been linked to HCV infection, we further analyzed their interactions on the risk of SSI in Table 5. Patients with DM but not HCV demonstrated a lower risk of SS (OR = 0.78, 95% CI = 0.72–0.84). On the contrary, patients with HCV but not DM had higher risks of SS (OR = 2.50, 95% CI = 2.11–2.95). The interaction between HCV infection and DM on the SS risk was not significant. However, HCA infection antagonisticly interacted with CAD and liver cirrhosis to modify the risk the SS (p<0.001). Patients with CAD but not HCV and patients with HCV infection but not CAD were significantly associated with higher risks of SS (OR: 1.28, 95% CI = 1.20–1.37 and OR: 3.00, 95% CI = 2.53–3.56, respectively). Similarly, the patients with liver cirrhosis but not HCV and the patients with HCV but not liver cirrhosis all showed higher risks of SS (OR: 2.74, 95% CI = 2,19–3.44 and OR:2.67, 95% CI = 2.30–3.11, respectively).

**Table 5:** Association of Sjögren’s syndrome with hepatitis C virus infection alone and interaction with comorbidities (diabetes, coronary artery disease, and liver cirrhosis).

		Cases	Controls	Crude OR (95% CI)	OR (95% CI)^a^
HCV alone	DM				
No	No	7,365	30,582	1.00 (reference)	1.00 (reference)
No	Yes	1,378	5,760	0.99 (0.93–1.06)	0.78 (0.72–0.84)^‡^
Yes	No	283	377	3.12 (2.67–3.64)^‡^	2.50 (2.11–2.95)^‡^
Yes	Yes	114	170	2.79 (2.19–3.54)^‡^	1.85 (1.43–2.39)^‡^
				Interaction p = 0.473	Interaction p = 0.759
HCV alone	CAD				
No	No	6,690	30,081	1.00 (reference)	1.00 (reference)
No	Yes	2,053	6,261	1.75 (1.39–1.56)^‡^	1.28 (1.20–1.37)^‡^
Yes	No	269	356	3.40 (2.89–3.99)^‡^	3.00 (2.53–3.56)^‡^
Yes	Yes	128	191	3.01 (2.41–3.78)^‡^	2.03 (1.60–2.59)^‡^
				Interaction p = 0.0004	Interaction p<0.0001
HCV alone	Liver cirrhosis				
No	No	8,599	36,143	1.00 (reference)	1.00 (reference)
No	Yes	144	199	3.04 (2.45–3.77)^‡^	2.74 (2.19–3.44)^‡^
Yes	No	328	467	2.95 (2.56–3.41)^‡^	2.67 (2.30–3.11)^‡^
Yes	Yes	69	80	3.63 (2.63–5.01)^‡^	3.28 (2.34–4.60)^‡^
				Interaction p<0.0001	Interaction p = 0.0003

^a^Adjusted for age, sex, geographic region, occupation, monthly income, hyperlipidemia, cancer, renal disease, coronary artery disease, chronic obstructive pulmonary disease, and liver cirrhosis (excluded CAD in HCV×CAD interaction model and excluded liver cirrhosis in HCV× liver cirrhosis interaction model)

* p<0.05

^†^ p<0.01

^‡^ p<0.0001

## Discussion

In this large-scale, population-based case-control study in a highly prevalent chronic HBV and HCV infection area, we observed a positive significant association between SS and chronic HCV infection. Sex-stratification analysis also revealed that HBV-infected men had a significantly higher risk of SS.

In 1992, Haddad et al. reported lymphocytic sialadenitis in 57% of HCV-infected patients and 5% of controls [10]; hence, the debate over the association between SS with HCV began two decades ago. Accordingly, numerous HCV-associated SS cases have been reported. SS has been suggested as one of the systematic autoimmune diseases most closely associated with HCV [29]. A higher prevalence of SS was noted in patients with HCV infection (25.9%) than in those with HBV infection (3.4%) in a Japanese study [9]. Ramos-Casals et al. reported that 13% of patients with chronic HCV infection in Spain also had SS [8]. A significant overall association between HCV infection and SS was observed in a recent meta-analysis [11]. There is increasing evidence from experimental [30], virological [31,32], and clinical studies [13,33,34] suggesting that HCV and SS may share some overlapping etiological characteristics. Ramos-Casals and his colleagues proposed the term SS “secondary to HCV” (SS-HCV) to implicate the development of SS in a particular subset of HCV patients [6]. Although there is no significant difference in any histological examination between the groups, SS-HCV tends to present at an older age and at a reduced female to male ratio when compared with primary SS patients without HCV. Ramos-Casals reported a reduced female:male ratio (3:1) and older age (58.3± 1.17 vs 52.7±0.85) of SS-HCV compared with primary SS patients [6]. In the study of Brito-Zeron et al., SS-HCV patients showed a reduced female:male ratio (5:1 vs 14:1) and an older age (mean age of 62.9 years) when compared with primary SS [5]. In our study, the female:male ratio and median age of the SS subjects were 6.7:1 and 55, respectively. Another Taiwanese group reported the female:male ratio of SS to be 7.9 [3]. In Taiwan, in order to be considered for a CIC for SS, patients must fulfill at least four criteria of the 2002 European classification for SS with at least one of the two mandatory criteria (positive salivary gland biopsy or anti-Ro/La antibodies). The application is then reviewed by rheumatologists commissioned by the Bureau of the NHI system for CIC eligibility and coded specifically with ICD-9-CM 710.2 for subsequent services. Although Ramos-Casals et al. proposed the term ‘‘SS secondary to HCV” for patients who fulfill the 2002 classification criteria for SS [6], we still feel confident with our data source. In addition, our data also showed that young HCV carriers carry a higher risk of SS when compared with non-HCV carriers.

SS-HCV patients also demonstrate lower frequencies of autoantibodies against Ro and La human ribonucleoproteins and complementaemia and a higher prevalence of cryoglobulinmic-related immunological markers including rheumatoid factor (RF) than primary SS patients without HCV [7,35]. However, the pathogenesis from autoantibody formation to the full clinical manifestation of HCV-associated SS is not well established. The proposed mechanism involves cross-reactivity between the HCV envelope and host salivary tissue or HCV envelope-mediated immune reaction against salivary glands [36]. Hence, Ramos-Casals et al. suggested that primary SS and SS-HCV are two separate processes [6]. However, whether HCV mimics primary SS or is directly responsible for the development of primary SS in a subset of patients remains controversial [35]. Further studies are needed to clarify this issue.

Some comorbidities have been associated with HCV in the literature including diabetes and CAD [37,38]. Hence, we further analyzed DM and CAD to investigate any possible interaction between HCV and the comorbidities that may have contributed to the association detected in this study between HCV and SS. Our results indicate that CAD and liver cirrhosis are independent risk factors of SS (OR = 1.28, 95% CI = 1.20–1.37 and OR = 2.74, 95% CI = 2.19–3.44, respectively) (Table 5). Interestingly, DM demonstrated an independent protective effect toward SS (OR = 0.78, 95% CI = 0.72–0.84). Further prospective studies are warranted to verify this finding.

In our study, we detected a higher odds ratio among younger patients with SS and HCV. We hypothesize that a gradual change in the HCV genotype and its interaction with environmental factors may underlie this observation. In Taiwan, the 1b HCV genotype has historically been the most prevalent in the general population, dwarfing the prevalence of subtype 2a [39]. The most suspected routes of transmission included illegal medical interventions, such as the use of nondisposable needles, the sharing of syringes and fluid for injection, and blood transfusion-related infections and may have been responsible for the spread of the 1b HCV genotype among older Taiwanese [39]. However, in recent years the 2a genotype has been observed with increasing frequency among younger individuals. These young people are often HIV-positive persons with a history of injection drug use [40]. One study demonstrated a high prevalence of HCV infection among HIV-infected intravenous drug users in Taiwan with a predominance of infection due to genotypes 1a, 6a, and 3a, instead of 1b [41]. 2a was a predominant genotype among acute hepatitis C among HIV-infected individuals in another matched case-control study in Taiwan [42].

HBV-related SS has rarely been investigated and remains controversial. Aprosin et al. suggested the involvement of the virus in the etiology of SS in early 1990 [14,43–46]. Marcos et al. reported that only 5 (0.83%) of 603 patients with SS tested positive for HBsAg, which hints at a null association between chronic HBV infection and SS [15]. In Taiwan, Chen et al. observed that 18 of 175 patients with SS were positive for HBsAg, revealing that SS has a significantly lower prevalence of HBV than the general population (10.3% vs. 17.3%, p < 0.001) [47]. In another Taiwanese study, the prevalence of anti-SSA and anti-SSB autoantibodies was lower in HBV carriers (1.9% and 0%) than in HCV carriers (12.8% and 9.7%). Both anti-SSA and anti-SSB antibodies are critical markers of SS [48]. In a study by Nagao et al., the prevalence of SS in patients with chronic HCV infection was significantly higher than that in patients with chronic HBV infection [9]. Recently, Ram et al. suggested that HBV infection protects against autoimmune disorders, including SS [14].

In our study, we clearly observed that patients with chronic HBV infection are at a lower risk for SS than those with chronic HCV infection. This may be explained through increased an increased risk of extrahepatic manifestations among HCV patients, involving renal, rheumatologic, dermatologic, as well as hematologic abnormalities [49]. Such extrahepatic manifestations are likely to arise from immunologically triggered mechanisms and virus invasion and replication. In HCV, lymphatropic character may explain the cause of HCV-associated extrahepatic manifestations [50] and may also explain why patients with HCV were observed to have a greater risk of SS than those infected with HBV.

A strength of this study is that it is the first to use a nationwide population-based data set to investigate the association between SS and chronic viral hepatitis. The statistical power offered by our large sample size enabled us to stratify our estimates of association by both sex and age, thus allowing us to provide the first sex and age specific estimates to the literature.

Several limitations of this study should be considered. First, it is possible that some patients with hepatitis were misclassified and were included in the control group. This may have occurred if they did not elect to seek medical treatment for their condition and thus did not receive a diagnosis. In any case, assuming that there is a causal association between viral hepatitis infection and SS, this misclassification would bias our estimate toward the null, thus leaving us with a more conservative estimate and greater confidence in the presence of the association.

Second, using ICD-9-CM diagnosis codes to identify patients with SS, HBV, and HCV infections, and comorbidities may not be as accurate as identifying patients in a clinical setting according to more standardized diagnostic criteria. However, the NHI Bureau has several self-policing mechanisms to better ensure for higher coding accuracy and quality of care. These include the scheduled random review of charts and claims along with patient interviews at every hospital with punitive measures being imposed for inconsistencies. The diagnostic accuracy among SS patients can be expected to be particularly high. SS patients are closely vetted prior to their inclusion in the CIC category due to the high economic burden incurred by the state which takes responsibility for the patient’s medical treatment. To minimize the possibility of enrolling patients with secondary SS in our case group, we excluded patients who were diagnosed with other autoimmune diseases before or after SS diagnosis.

Third, while our use of the CIC category enhanced our case validity, our utilization of two data bases may have biased the results of our analysis. By only selecting CIC cases, we may have been able to avoid recruiting patients with sicca syndrome, which is only defined by symptoms, and solely recruit SS cases that require rheumatologists to assess specific diagnostic criteria. However, we used the LHID2000 to select our controls. The stringent diagnostic criteria required for the CIC program produces a difference in the severity of disease found among CIC patients and the general population in the LHID2000. Thus, it is possible that the LHID2000 contained less severe version of SS that were not included in the CIC program. If this were the case, some of our controls may have had mild versions of SS which would have worked to bias our results towards the null and produce a more conservative estimate.

Fourth, some may argue that HBsAg and anti-HCV are not reliable metrics for defining chronic viral hepatitis infections. However, in our previous study, the predictive value of HBsAg testing alone was 97% among 367 adults born prior to the national HBV vaccination [51]. In addition, to further increase the diagnostic accuracy, this study only considered subjects to have HBV and HCV if they received at least three out-patient diagnoses or at least one in-patient diagnosis.

Fifth, nearly all the residents of Taiwan are of Han Chinese ethnicity and our results should be generalized with caution. Due to epidemiological, demographic, and cultural differences, it is likely that the transmission route of viral hepatitis differs between locations and peoples.

Sixth, it is possible that viral hepatitis patients were more likely to be diagnosed with SS purely on account of their increased exposure to the medical community. However, on account of the low out-of-pocket costs, lack of barriers to specialist care, and convenient access across the country provided by the Taiwanese national insurance program, Taiwanese readily seek medical care in the event of any discomfort, thus making this type of bias highly unlikely.

Seventh, laboratory data were unavailable in the claims records; therefore, we could not analyze the risk factors for SS in detail. Finally, according to both prior research and the results of this study presented in Table 5, it is evident that liver cirrhosis is an independent risk factor for SS. While we have statistically adjusted for the effect of liver cirrhosis in each of the analyses performed in this study, it remains possible that our results suffered from residual confounding.

## Conclusions

We found evidence indicating that chronic HCV infection associates with SS. However, our results also indicate that patients with chronic HBV infection are at a lower risk for SS when compared with those with chronic HCV infection. Additional studies are warranted to apply our findings to other geographical regions or races, and for clarifying the biological mechanisms underlying the associations detected in this study.
