# Correction: Association of Sjögren's Syndrome in Patients with Chronic Hepatitis Virus Infection: A Population-Based Analysis

**DOI:** 10.1371/journal.pone.0164911

**Published:** 2016-10-17

**Authors:** Chih-Ching Yeh, Wen-Chang Wang, Chien-Sheng Wu, Fung-Chang Sung, Chien-Tien Su, Ying-Hua Shieh, Shih-Ni Chang, Fu-Hsiung Su

There is an error in affiliation 8 for authors Ying-Hua Shieh and Fu-Hsiung Su. Affiliation 8 should be: Department of Family Medicine, School of Medicine, College of Medicine, Taipei Medical University, Taipei, Taiwan.
